# Communication about sexual orientation and gender between clinicians, LGBT+ people facing serious illness and their significant others: a qualitative interview study of experiences, preferences and recommendations

**DOI:** 10.1136/bmjqs-2022-014792

**Published:** 2022-08-31

**Authors:** Debbie Braybrook, Katherine Bristowe, Liadh Timmins, Anna Roach, Elizabeth Day, Paul Clift, Ruth Rose, Steve Marshall, Katherine Johnson, Katherine E Sleeman, Richard Harding

**Affiliations:** 1 Cicely Saunders Institute of Palliative Care, Policy & Rehabilitation, Florence Nightingale Faculty of Nursing, Midwifery & Palliative Care, King's College London, London, UK; 2 Columbia Spatial Epidemiology Lab, Columbia University Mailman School of Public Health, New York, New York, USA; 3 Patient and Public Involvement member, London, UK; 4 Patient and Public Involvement member, Brighton, UK; 5 Palliative Care, Cicely Saunders Institute, King's College Hospital NHS Foundation Trust, London, UK; 6 School of Global, Urban and Social Studies, RMIT University, Melbourne, Victoria, Australia

**Keywords:** communication, healthcare quality improvement, health professions education, patient-centred care, qualitative research

## Abstract

**Background:**

Healthcare organisations have legal and ethical duties to reduce inequalities in access to healthcare services and related outcomes. However, lesbian, gay, bisexual and/or transgender (LGBT+) people continue to experience and anticipate discrimination in health and social care. Skilled communication is vital for quality person-centred care, but there is inconsistent provision of evidence-based clinician education on health needs and experiences of LGBT+ people to support this. This study aimed to identify key stakeholders’ experiences, preferences and best practices for communication regarding sexual orientation, gender identity and gender history in order to reduce inequalities in healthcare.

**Methods:**

Semistructured qualitative interviews with LGBT+ patients with serious illness, significant others and clinicians, recruited via UK-wide LGBT+ groups, two hospitals and one hospice in England. We analysed the interview data using reflexive thematic analysis.

**Results:**

74 stakeholders participated: 34 LGBT+ patients with serious illness, 13 significant others and 27 multiprofessional clinicians. Participants described key communication strategies to promote inclusive practice across three domains: (1) ‘Creating positive first impressions and building rapport’ were central to relationship building and enacted through routine use of inclusive language, avoiding potentially negative non-verbal signals and echoing terminology used by patients and caregivers; (2) ‘Enhancing care by actively exploring and explaining the relevance of sexual orientation and gender identity’, participants described the benefits of clinicians initiating these discussions, pursuing topics guided by the patient’s response or expressed preferences for disclosure. Active involvement of significant others was encouraged to demonstrate recognition of the relationship; these individual level actions are underpinned by a foundation of (3) ‘visible and consistent LGBT+ inclusiveness in care systems’. Although participants expressed hesitance talking about LGBT+ identities with individuals from some sociocultural and religious backgrounds, there was widespread support for institutions to adopt a standardised, LGBT+ inclusive, visibly supportive approach.

**Conclusions:**

Person-centred care can be enhanced by incorporating discussions about sexual orientation and gender identity into routine clinical practice. Inclusive language and sensitive exploration of relationships and identities are core activities. Institutions need to support clinicians through provision of adequate training, resources, inclusive monitoring systems, policies and structures. Ten inclusive communication recommendations are made based on the data.

**WHAT IS ALREADY KNOWN ON THIS TOPIC**

- Healthcare organisations have legal and ethical duties to reduce inequalities in access to healthcare services and related outcomes, but lesbian, gay, bisexual and/or transgender (LGBT+) people continue to report discrimination and exclusion in health and social care and have higher risk of some serious illnesses.
- Skilled communication is central to person-centred care, but the health needs and experiences of LGBT+ people are not consistently included in clinician curricula, and there is an absence of evidence-based communication guidance for clinicians on working with LGBT+ people.

**WHAT THIS STUDY ADDS**

- Clinicians want to provide LGBT+ inclusive care but report a lack of adequate training, inconsistent support at organisational level and anxiety around how to talk about sexual orientation and gender identity. This can be exacerbated when caring for patients from some sociocultural and religious backgrounds.
- LGBT+ people facing serious illness find it easier to be open when assumptions are avoided, non-verbal signals attended to, direct questions about identity are made relevant to care and organisations demonstrate LGBT+ inclusivity.

**HOW THIS STUDY MIGHT AFFECT RESEARCH, PRACTICE OR POLICY**

- Our 10 evidence-based recommendations can support clinicians and healthcare organisations to deliver LGBT+ inclusive care in routine practice and may help to achieve national policy and monitoring standards. Full guide here: www.kcl.ac.uk/nmpc/assets/research/projects/abc-lgbt-inclusive-communication.pdf

## Introduction

Data from 27 countries revealed that 8% of adults have a minority sexual orientation, and 1% identify as trans or non-binary.^1^ Despite legal advances to protect LGBT+ people from healthcare discrimination in many countries,^2^ experience and anticipation of discrimination within healthcare continue to be concerns, which can delay seeking health services.^3–11^ The acronym LGBT+ refers to lesbian, gay, bisexual and/or transgender people, and anyone who considers themselves to have a minority sexual orientation, gender identity or gender history (the relationship between gender identity and sex assignedat birth).

Experience of discrimination among LGBT+ people is linked to higher incidence of serious illness (eg, cancer, cardiovascular disease and respiratory disease),^12^ poorer health outcomes,^13–16^ more risky health behaviours (eg, alcohol and tobacco consumption) and difficulties accessing healthcare.^15 17–25^ When seriously ill LGBT+ people feel unable to share important aspects of identity, the impact can be devastating.^6 26 27^

Competent communication is vital for LGBT+ inclusive care,^28–30^ building trusting relationships, informed decision making and person-centred care.^31^ The WHO identifies person-centred care as one of its seven domains of quality, focusing on the individual so that care ‘responds to individual preferences, needs and values’ including social circumstances and lifestyle.^32 33^ Poor communication and assumptions made by clinicians about patients’ gender and sexual orientation undermine clinical relationships, leading to disengagement and loss of trust. Specifically, heteronormativity (the assumption that being heterosexual is the ‘normal’ sexual orientation) and cisnormativity (the assumption that having a gender identity that aligns with sex assigned at birth is the ‘normal’ gender identity) are pervasive and damaging.^34 35^

To improve LGBT+ inclusive communication and care, it is vital to understand challenges, preferences and potential benefits for all key stakeholders—patients, significant others and clinicians.^5 6 36^ Although clinicians are often overlooked in LGBT+ health research,^36–38^ their participation is essential to understand how prior clinical experiences shape communication behaviours and inform feasible and acceptable communication strategies. This study aimed to investigate experiences and preferences regarding communication about sexual orientation, gender identity and gender history in the context of serious illness (where involvement of significant others and person-centred communication and decision making are vital) and to identify best practice to inform evidence-based recommendations for clinicians and educators relevant to all clinical scenarios.

## Methods

### Design

This national, qualitative interview study sampled three populations in England to optimise the feasibility and acceptability of recommendations: (1) LGBT+ people with serious illness (patients), (2) their informal caregivers (significant others) and (3) clinicians. The study is reported in accordance with the COnsolidated criteria for REporting Qualitative research checklist.^39^

### Procedure

#### Team

Our team comprised: clinicians (medical consultant, social worker); five experienced qualitative researchers; a sociolinguist; a psychological scientist; a sociologist; three experts in healthcare intervention and improvement; and six researchers experienced in LGBT+ health research.

#### Patient and public involvement

Three LGBT+ patient and public involvement (PPI) team members were integral to the study. PC, ED and RR attended ethics committee meetings and steering group meetings, supported recruitment, contributed to analysis and recommendation development, wrote lay summaries and presented findings at dissemination events.

#### Inclusion criteria

LGBT+ patients with serious illness: ≥18 years old, self-identified as LGBT+, living with serious illness (a condition that carries a high risk of mortality, negatively impacts quality of life and daily function and/or is burdensome in symptoms, treatments or caregiver stress).^40^

Significant others: ≥18 years old, ‘unpaid, informal providers of one or more physical, social, practical and emotional tasks. In terms of their relationship to the patient, they may be a friend, partner, ex-partner, sibling, parent, child or other blood or non-blood relative’.^41^

Clinicians: doctors, nurses and social workers employed by the National Health Service (NHS) with experience caring for patients with serious illness.

#### Exclusion criteria

Patients and significant others were excluded if they lacked cognitive capacity to give informed consent or were too frail/unwell or distressed to participate.

#### Sampling and recruitment

Patients and significant others were recruited nationally through LGBT+ organisation mailing lists, newsletters and social media platforms and through clinical teams (hepatology, HIV, palliative care, pulmonology and renal) at two hospitals and one hospice in England. Those who self-referred were appraised against the inclusion and exclusion criteria by the researcher and discussed with the clinical site lead as required. Purposive sampling criteria were applied to patients: diagnosis, age, ethnicity, gender modality and sexual orientation. Significant others could participate without the patient entering the study. We sampled professions (doctors, nurses, social workers) anticipated to be consistently involved across serious illnesses. Recruitment, data collection and analysis were conducted iteratively to inform subsequent sampling. We continued to conduct interviews until we reached pragmatic saturation, as relevant to the study aims and objectives.^42 43^

#### Data collection and analysis

Semistructured interviews^44^ were conducted using a topic guide informed by systematic reviews,^5 26^ empirical work^6^ and revised with steering group and PPI members. DB conducted the interviews in a location of the participant’s choice (including home, workplace, public setting, eg, cafes, libraries, interviewer workplace and remotely for some clinicians). The researcher was known professionally to two participating clinicians. Following demographic questions, participants were asked to describe how sexual orientation and gender identity/history impacted care experiences, challenges and opportunities in communication, examples of good and poor practice, preferred approaches to incorporating these areas into healthcare communication, views on identity monitoring and recommendations (topic guide – online supplemental 1). Field notes captured context and reflections and informed future interviews. Interviews were audio recorded, transcribed verbatim, pseudonymised and uploaded to NVivo V.12 for analysis.

10.1136/bmjqs-2022-014792.supp1 Supplementary data

Reflexive thematic analysis^45^ was conducted through data familiarisation and immersion, coding, developing initial themes, then reviewing developing and defining/naming themes. Initial coding was inductive and subsequent coding informed by theories of person-centred and holistic care.^46 47^ DB read and coded all transcripts. RH read five early transcripts and KB and RH met with DB to discuss coding and theme generation to inform ongoing analysis. The draft and final coding frame were reviewed by all authors for interpretation.

This work is positioned within a critical realist paradigm, where our interpretations of reality are shaped by our standpoint.^48 49^ Our research and PPI team includes individuals who identify as lesbian, gay, bisexual, queer, heterosexual, non-binary, women, men, trans and cisgender.

Our previous work with LGBT+ people demonstrates exclusion and discrimination in care settings^5 6^ and has informed work of regulators of healthcare services^50^ and government inquiries to support improvements in care for LGBT+ people.^51^

## Results

### Sample characteristics

Seventy-four participants were interviewed (November 2018–November 2019): n=34 patients, n=13 significant others, n=27 clinicians (see table 1). All interviews with patients and significant others were conducted face to face (median duration 113 min, range 55–233 min) and one to one (except one patient and significant other who participated as a dyad). In two interviews, a significant other was present in the room but did not participate. Eighteen clinicians were recruited from hospitals and nine from hospices (median duration 104 min, range 50–190 min). Eight clinician interviews were conducted remotely. After receiving study information, 24 people chose not to participate: too unwell (four patients), uninterested (four patients), lack of time (two patients, three clinicians) and reason unknown (eight patients, three clinicians).

**Table 1:** Sample characteristics

		Qualitative interview study participants n=74					
		LGBT+ patients n=34		Significant others n=13		Clinicians n=27	
		n	%	n	%	n	%
**Ethnic group**	White: English, Welsh, Scottish, Northern Irish, British, Irish, other white background	**28**	82	**12**	92	**23**	85
	Mixed ethnicity/multiple ethnic groups	**4**	12	**1**	8	**2**	7
	Asian/Asian British					**1**	4
	Black/African/ Caribbean/black British	**2**	6			**1**	4
**Age group (years)**	20–39	**11**	32	**1**	8	**8**	30
	40–59	**13**	38	**10**	77	**19**	70
	60–79	**10**	29	**2**	15		
**Sexual orientation (self-described)**	Gay (man, woman or non-binary person)	**21**	62	**7**	54	**9**	33
	Lesbian	**6**	18	**2**	15	**2**	7
	Queer	**3**	9				
	Bisexual	**1**	3	**1**	8	**4**	15
	Pansexual	**1**	3				
	Homosexual	**1**	3				
	Heterosexual			**1**	8	**12**	44
	Straight	**1**	3				
	Fluid or undefined			**2**	15		
**Gender identity and history**	***All men***	***20***	*59*	***6***	*46*	***13***	*48*
	Transgender men	**1**	5			­	­
	Cisgender men	**19**	95	**6**	100	­	­
	***All women***	***9***	*26*	***7***	*54*	***14***	*52*
	Transgender women	**2**	22			­	­
	Cisgender women	**7**	78	**7**	100	­	­
	***All non-binary people including genderqueer, gender-fluid and gender non-conforming people***	***5***	*15*			***­***	***­***
	Non-binary person assigned female at birth	**3**	60				
	Non-binary person assigned male at birth	**2**	40				
**Relationship status**	Single	**17**	50	**3**	23	***­***	***­***
	In relationship	**17**	50	**10**	77	***­***	***­***
**Serious illness category**	Cancers	**6**	18			***­***	***­***
	Non-cancers (eg, gastro, liver, lung, neuro, renal)	**9**	26	**6**	46	­	­
	Comorbidities	**19**	56	**7**	54	***­***	***­***
**Home/workplace**	Greater London	**27**	79	**11**	85	**21**	78
	England (outside London)	**7**	21	**2**	15	**6**	22
**Job title**	Social worker	***­***	***­***	***­***	***­***	**7**	26
	Nurse	***­***	***­***	***­***	***­***	**13**	48
	Doctor	***­***	***­***	***­***	***­***	**7**	26

Note that sometimes percentages do not total to 100 due to rounding to the nearest whole number

### Findings

#### Overview

Three main themes were generated: (1) creating positive first impressions and building rapport; (2) enhancing care by actively exploring and explaining the relevance of sexual orientation and gender identity; and (3) visible and consistent LGBT+ inclusiveness in care systems. Exemplary quotes are presented for each theme, with additional quotes from across the populations sampled in online supplemental 2.

10.1136/bmjqs-2022-014792.supp2 Supplementary data

#### Theme 1: creating positive first impressions and building rapport

Some routinely used terminology and practices can feel excluding to LGBT+ people. Using appropriate terminology was important for all stakeholder groups. For clinicians, this was underpinned by fear of offending. Although positive experiences were described, many service users shared negative experiences, often linked to incorrect assumptions, which caused distress and unnecessary emotional labour.

[T]hey’ve quite often asked if [my partner’s] my husband or just assume, err ‘Oh your husband let me in’ and I said ‘Oh I haven’t actually got a husband’ (laughter) and they said ‘Oh the man.’ I say ‘No that’s not a man, that’s a lady.’ Yeah, so I’ve had to explain that situation quite a few times. (Patient, lesbian female, cisgender, in her 50s)

Incorrect assumptions about LGBT+ identities negatively impact relationships with clinicians and force LGBT+ people to decide whether to correct the clinician or allow the assumption to stand. Either choice can provoke anxiety and undermine trust.

If I’d said, ‘And, is your partner working? Is he working?’ to a woman, […] I mean I’m very conscious not to, […] that would be one of those hiccups because the patient might just choose to answer, ‘No they’re not,’ and avoid giving gender but would take that mental note of [pause] you know, ‘She’s assumed I’m heterosexual’ you know and whatever emotion that stirs up for them. The ‘Do I— [pause] Is it safe to disclose or not?’ You know that, that constant ‘Do I have to come out? Do I want to come out?’ (Nurse, in her 50s)

In response to these challenges, participants across the stakeholder groups described simple ways to achieve inclusive communication.

##### Using neutral language

All three populations advocated avoiding heteronormative and cisnormative assumptions. An effective approach was to use neutral language until relationships and identities are established. For example, using neutral references to gender (eg, ‘they’) avoids assumptions about sexual orientation or gender identity.

… a lot of the time the hospital sends letters to the GP and it’ll be like ‘I met this lovely young lady’ and that just *really* annoys me […] that’s not who I am, and I feel like it springs up this image in people’s minds that isn’t me. So that kind of makes me uncomfortable. Those certain phrases whereas if they were just gender neutral I’d be a lot better with it. (Patient, queer non-binary person, assigned female at birth, in their 20s)

##### Listening and echoing terminology

Within a consultation, a clinician should use the words that patients and significant others have chosen to describe themselves and their relationships.

[U]sing the language that the patient uses, so not changing say pansexual into bisexual or, you know not making assumptions […] and if you’re gonna note it down, note it as that, if you’re gonna carry on a conversation then carry it on like that. (Patient, pansexual male, transgender, in his 20s)

Not paying attention to language can be distressing, as it implies the clinician either is not listening, or objects in some way.

[My partner] gets frustrated when people, when she says ‘partner’ and they say ‘boyfriend’ or ‘husband’ or— And I’m like ‘No it’s my girlfriend.’ Or sister, or— I’m everyone *but* ‘girlfriend’ when I’m there. (Significant other, lesbian female, cisgender, in her 30s)

#### Considering non-verbal signals

Awareness of non-verbal signals is vital to LGBT+ inclusiveness. Shifts in body positioning or facial expressions after a disclosure can be interpreted as discomfort or negativity.

[W]hen I say, ‘lesbian,’ […] I’ll look for a little micro-expression behind the eyes, a twitch or—, to see how sensitive they are to it. […] I can almost read, okay, you’ve taken that on the chin, I respect that. […] you didn’t make me feel bad. (Patient, gender non-conforming lesbian, assigned female at birth, in her 60s)

Tone, pitch and volume of speech also warrant attention, as they can be suggestive of clinicians’ views surrounding a topic.

[I]t’s not necessarily what they say, but just their tone, […] they react as if they’ve heard it all before. It’s when they ask as if it’s nothing as well […] which makes me feel more relaxed about answering it honestly. (Patient, gay male, cisgender, in his 20s)

#### Theme 2: enhancing care by actively exploring and explaining the relevance of sexual orientation and gender identity

Participants gave divergent views on discussing LGBT+ identities. Sensitive exploration of preferences for disclosure is an important first step.

[I]f someone’s in a relationship that they haven’t traditionally been able to be open about, then that may affect how they can communicate with healthcare, or had bad experiences with healthcare professionals in the past, or had assumptions or more old-fashioned societal rules. (Doctor, in her 40s)

While discussions about LGBT+ identities were viewed as appropriate for holistic, person-centred care, some participants saw these as sensitive topics, and clinicians sometimes avoided initiating discussions.

[Y]ou do ask sometimes like ‘How would you describe your sexual orientation?’ There are times maybe where I miss it out because I’m not sure how to ask. […] I know this is maybe not great, but where you kind of think someone might not be straight, you’re more likely to ask. But that’s not good practice. (Doctor, in his 30s)

Some clinicians considered broaching these topics a potential threat to relationships and rapport, particularly with limited time to build relationships.

[Y]ou do in hospice care but you don’t always outside of that, get that time to build a relationship where people will trust you. So, it can be very hidden and that means that people may not always get appropriate support because they haven’t talked to you about that. (Social worker, in her 50s)

While apprehension may cause avoidance of discussion, incorrect assumptions and absence of early candid discussions create subsequent difficulties for all stakeholder groups.

[T]he longer you leave it the harder it is to ask and the harder it is to tell definitely. (Significant other, gay female, cisgender, in her 40s)

##### Communicating relevance of LGBT+ matters as part of high-quality, person-centred care

There was widespread agreement that explaining the relevance of questions about LGBT+ identities can increase acceptability.

[I]t should come from the healthcare professionals because in a way that would inform their treatment of the person […], but also it would inform any decisions that they might make on behalf of their patient […] I think a lot of people probably in a similar way to me, probably wouldn’t necessarily know if it was safe to bring it up themselves, or whether they’re just gonna get dismissed or scoffed at. (Patient, queer non-binary person, assigned female at birth, in their 20s)

When asking patients questions about LGBT+ identities, providing a rationale for the questions, an opportunity not to respond if preferred and asking permission to record information enables informed disclosures.

I would always make it clear why I’m asking […] ‘If I’m going to look after you this is something I might need to know, to help me look after you’ […] I don’t want anyone to think that sometimes we’re prying and asking things we don’t need to know about or I might you know give the person the option of actually not answering the questions. (Doctor, in his 50s)

##### Respecting gender

Use of incorrect pronouns is distressing for the individual and impacts on relationships with clinicians.

It is better to ask me ‘What pronouns do you use?’ rather than call a woman a man, or address a woman with masculine pronouns […] any sort of doubt at all, don’t be afraid to ask which pronouns you’re using. (Patient, bisexual female, transgender, in her 50s)

Being referred to as ‘trans’ immediately discloses a person’s gender history. As such, clinicians need to understand how to frame questions about gender history where relevant to the care they provide.

[P]eople sort of ask ‘When did you transition?’ […] it’s a common way to talk about it but it is essentially meaningless. Because, my transition started long before any form of medical intervention happened and its gonna carry on for the rest of my life. […] Do you need to know when I started on hormones? Do you need to know about any surgeries that I’ve had? Like these are the things that I think you are trying to ask but actually you need to ask them specifically, because they are scattered over a number of years. (Patient, queer non-binary woman, transgender, in their 30s)

Additional sensitivity and attention are required in discussions about anatomy in the context of treatment.

[I]f that person has said to you ‘No I identify, as female’ […] and they’re talking about their genitals then there is nothing wrong with saying, you know ‘her penis’. (Patient, pansexual male, transgender, in his 20s)

##### Including significant others and sexual orientation appropriately

Participants across the samples noted the importance of identifying significant others.

If I haven’t met them but I’ve met the patient […] I’ll say ‘Who’s most important to you? Can I contact them to offer support?’ and most people are grateful for you to do that. (Social worker, in her 40s)

Understanding the depth and nature of a relationship helps clinicians to appropriately include significant others in decision-making and care.

[T]here are a lot of people who aren’t able to or don’t feel comfortable being open about their relationships […] and that may affect their symptom management or their experience of end-of-life care or the ability of their loved one to be there and supportive to them. (Doctor, in her 40s)

Both language and behaviours are important in involving significant others appropriately.

[M]y partner’s like [pause] like the kitchen team on the ward would […] try and get into conversation, ask how *they* are. Really *include* them, and so would, like the *nurses* and everyone […] *talking*, directly to them. I’ve never really had that even with sort of family members, even if they sit with me in consultation, *I'll* just be focused on. So, I’ve never had issues with partners or anything, ever. (Patient, lesbian female, cisgender, in her 20s)

LGBT+ patients who commence new relationships during their care may also feel anxious about involving new partners if clinicians are unaware of their sexual orientation.

I don’t have a long-term partner. And when I was dating, and I was trying to take people with me, there always was that thing of like, this is something that I’m going to have to add to my appointment. You know, that extra level of stress. Yeah. Because we’ve never had that real, like, declarative conversation - ‘This is me’ - coming out to the whole department. (Patient, gender-fluid gay person, assigned male at birth, in their 30s)

Some participants felt it beneficial to explicitly include sexual orientation in care-related discussions at the outset.

[S]exual orientation, I feel like, yeah, they should know this about me. […] If it’s recorded at that stage, at the beginning […] and then they’ve got it on record, then it can be acknowledged because then you know you’ve given that information. (Patient, gay male, cisgender, in his 20s)

##### Considering the environment and who else is present

Many participants described the artificial nature of drawing curtains around patients in a ward and the potential threat to confidentiality and personal safety.

I think environment is really key, isn’t it? So, like, clinic room; you’re on your own. Curtains on a ward; not soundproof. […] *I* might be very cool about it, and *try* and be very cool about it, but you don’t necessarily want to out someone to a ward of strangers that they are staying with. [pause] I think that puts them in a very vulnerable position [pause] really. Um, for any, kind of, orientation. And then, I think *particularly* for trans individuals. That’s a *huge* problem [pause] really. (Doctor, in his 40s)

Stakeholders also raised the importance of who is with a patient and recognising that patients may not have shared their sexual orientation or gender history with friends or family members present.

I wouldn’t say it outside of the hospital. I wouldn’t say it to my workmates, I wouldn’t say it to my family although I—, some of them may know already […] but they’ve never approached me directly. […] I would speak about it with health professionals yeah but not outside. (Patient, gay male, cisgender, in his 60s)

#### Theme 3: visible and consistent LGBT+ inclusiveness in care systems

Participants valued visible, clear and consistent LGBT+ inclusiveness within health systems and resources, while clinicians noted the lack of specific training within curricula.

[O]ne section of a one hour lecture at medical school in the genito-urinary medicine block probably where certain elements of sexual practice were touched upon. No. No more than that. […] I haven’t sought out courses in that field, so I have no other formal training. (Doctor, in his 40s)

##### Standardising the approach to LGBT+ related discussions

Routine inclusion of sexual orientation and gender identity in care processes may provide a structure to support clinicians.

I think when you *aren’t* meeting lots of LGBT+ people then, it’s *not* normalised to ask it, and then it becomes scary and it becomes awkward, and quite a lot of that is you as a health professional, and actually, normalising it within the NHS processes as questions that *everybody* is going to ask takes away the fear of offending anybody. (Doctor, in her 40s)

The use of routinely applied questions may reassure service users that they are not being selected for, or omitted from, such discussions.

I think pronouns are quite often used as a, a sort of Trojan horse to ask someone about their gender history […] ‘Ooh I think this person might be transgender.[…] we’ll ask pronouns,’ and it is very noticeable when you walk into a room and nobody asks anybody’s pronouns and then they go up to the trans person and they want to be all woke and pretend they’re a good ally; ‘What are your pronouns?’ ‘My pronouns are this’ and it’s like, cool, I can see what you’re doing there but also you were just saying like, ‘Hi trans person’. (Patient, queer non-binary woman, transgender, in their 30s)

Assumptions regarding social, cultural and religious background were identified as barriers to LGBT+ inclusive practice. Clinicians and service users described hesitation when considering discussion of sexual orientation and gender identity with people from some demographic groups, including older people, people with religious beliefs and people from black, Asian and ethnic minority backgrounds.

I know that culturally, you know sort of black and ethnic minority people, it’s very difficult for them to be gay, and it can be very, very taboo. […] we have a lot of African patients, and I would find that quite a difficult question. […] I would *do* it and I would *ask* because […] if they've got someone who appeared to be a same sex partner I’d want to know. […] you can't kind of just let that influence your practice and *not* do it, and be like ‘Oh okay, *my* belief is that African people find it very difficult to talk about being gay’, and actually they might not. They might be a person who’s really fine with it. (Nurse, in his 40s)

##### Establishing inclusive processes

For participants who were comfortable sharing such information, standardised recording was considered useful for avoiding repetition, assumptions and mistakes.

So it should be ‘Are you gay? Are you straight?’ ‘Yes’ that’s it. Just get it out in the open […] you don’t want to have to go through the thing every time so just get it on the record. (Patient, homosexual male, cisgender, in his 60s)

Transgender participants described systems’ inability to accurately capture gender identity (titles, names and pronouns) without amending sex on their medical record.

I said ‘Can you please call me under the name Karen (surname)?’ ‘Okay fine’ and they put a note on the notes […] and the receptionist comes to call me ‘(birth name)’ […] I totally ignored her and they called *three times* and I ignored them every time and then after about 10 or 15 seconds, […] I stood up, I walked over to her and said ‘I did *ask*’. (Patient, straight female, transgender, in her 60s)

However, service users and clinicians recognised that changing recorded sex may impact on the individual’s ability to receive appropriate healthcare resulting in a conflict between use of affirming communication and access to appropriate healthcare.

[T]he NHS has a huge, a blind spot in regards to those because those *calls* for scanning and screening are based on your gender in your documents. So, if you *change* your gender identity to female because you’re a trans woman, you won’t get *called* for prostate screening. (Doctor, in his 40s)

Several participants described concerns regarding information sharing within clinical teams, and the need to explain who can see that information.

[H]e was a gay man in a same-sex relationship and he didn’t want that disclosed. […] He talked to me about that but he didn’t want anyone in the team to know because he’s worried about how they might view him and he was very worried about receiving services. (Social worker, in her 50s)

##### Markers of inclusiveness

Markers of inclusivity (eg, LGBT+ inclusive images, indicators of relevant training delivered, inclusive policies, lanyards or badges in relevant colours) were reassuring to patients, signifying that clinicians welcome discussion of these aspects of identity.

[M]aybe just having a little bit of *visibility* around hospital *for* patients. […] you have all the pictures up of all the staff who’ve done really well, like, advertising the hospital. Like, one of them could have a rainbow lanyard on. I don’t know if they *do*. I haven’t actually seen. Like, just something *small* like that. You know. ‘‘Cause it only takes something small for people to think ‘Cool, that’s me [pause] That’s *me* there.’ And like, when I come next month, I know that there’s people here who are gonna *understand* me, on a level that I *need* you know, when you’re in care. (Patient, gay genderqueer person, assigned female at birth, in their 20s)

##### Recommendations for LGBT+ inclusiveness in clinical communication

The recommendations based on the data are presented in box 1 in line with the three main themes from study findings.

Box 1 **Recommendations for LGBT+ inclusiveness in clinical communication**

**Creating positive first impressions and building rapport**

1. *Use neutral language* , *such as neutral pronouns or neutral terms for significant others* . Neutral pronouns such as they/them, and neutral terms like ‘partner’ or ‘person’.
2.
*Use the words your patients use to describe themselves and significant others* . If your patient refers to a significant other as ‘they/them’ or as a ‘partner’ or ‘friend’ use the same words.
3.
*Consider the messages your non-verbal signals might send* . When discussing sexual orientation and gender identity, be mindful of the impact of potential non-verbal signals of discomfort. For example, your facial expression or volume/tone of voice may be suggestive of surprise/disapproval, or physical expressions such as shifts in posture/eye contact may be suggestive of discomfort.

**Enhancing care by actively exploring and explaining the relevance of sexual orientation and gender identity**

1.
*Create a safe space by making your questions about sexual orientation and gender relevant to care* . Explicitly state why you are asking these questions, and give an option not to answer, so that patients can make an informed choice. This will vary depending on clinical specialty, for example, you may be asking to ensure they are receiving required screening invitations, or because you want to ensure patients’ significant others are being included appropriately.
2.
*Respect gender* . Routinely introduce questions about gender identity and pronouns into your practice so you provide opportunity for patients to share and ensure you refer to them correctly. You could try saying ‘I want to make sure we are using your names and pronouns correctly. My name is XXXX and my pronouns are YYY/yyy. What about you?’. Only ask about gender history in private, using specific, justified questions.
3.
*Incorporate significant others and sexual orientation appropriately* . Ask about significant others inclusively, with neutral language. You might say ‘So I can look after you the best I can, can you tell me who’s important to you?’. If asking about partners or spouse, avoid gendered terms (such as wife or boyfriend). Instead, you could ask ‘Do you have a partner?’.
4.
*Consider your surroundings and who else is there* . Ensuring that patient preferences are known before discussing sexual orientation and gender identity where other people, including significant others, might overhear is vital.

**Visible and consistent LGBT+ inclusiveness in care systems**

1.
*Standardise how LGBT+ related discussions are approached* . Asking LGBT+ related questions consistently, regardless of social, cultural, religious and political backgrounds, makes discussions easier and more acceptable.
2. *Having LGBT+inclusive processes and systems in place*. Digital clinical records are central structures which, when designed inclusively, can bolster care for LGBT+ people. With patients’ consent, recording sexual orientation and gender identity in clinical records avoids repetition and prevents mistakes in correspondence.
3.
*Visual markers of LGBT+ inclusiveness* . LGBT+ inclusive policies, inclusive organisational materials and indicators of relevant training received should be in place, visible and easily accessed. Wearing a badge/lanyard in LGBT+ related colours shows inclusiveness and offers additional comfort.

## Discussion

### Principal findings

Our data demonstrate that inclusive discussions about identities and relationships, in an appropriate environment with explanation of relevance to care, may assist clinicians to deliver appropriate person-centred care. Formal training, inclusive assessment and monitoring systems, appropriate policies and visible demonstrations of inclusivity are central to this. While social, cultural and religious background can influence willingness to initiate and engage in discussions about sexual orientation and gender identity, avoidance of these discussions may risk compounding existing intersectional inequalities. Our 10 evidence-based recommendations offer a clear approach to realise the intentions of health equality and equity policy.

### Strengths and limitations of the study

This is the largest published qualitative study to specifically explore the experiences and preferences for communication about sexual orientation, gender identity and gender history in healthcare. It is the first to incorporate perspectives of clinicians, LGBT+ patients with diverse identities and illnesses and their significant others. By targeting serious illness, we aimed to explore communication preferences for person-centred care (a central tenet of all quality healthcare)^33^ and when involving significant others. Our recommendations are intended to foster trust^52 53^ between service users and clinicians at every interaction, thus facilitating care that accounts for preferences, needs and values at times of heightened need and also during usual care provision.

Although the eight transgender and/or non-binary patients and three bisexual/pansexual service users provided diverse depth of insight, we acknowledge that our sample cannot fully describe the breadth of trans/non-binary and bisexual/pansexual experience. Also, despite efforts to recruit participants across England and the UK, the majority of service users (38/47) and clinicians (21/27) were based in Greater London, which has a comparatively large LGBT+ population. We anticipate different experiences in less urban areas. We recruited 7/47 service users having black, Asian or ethnic minority backgrounds, which is a good participation rate in LGBT+ health research.^14^ Our findings are drawn from patients in England, and although this is an ethnically diverse country, recommendations should be appraised for relevance by clinicians working in different settings.

### Other related studies

Previous studies have described important tenets of inclusive clinical communication,^54 55^ many of which are confirmed by our study. Our study contributes to the evidence by identifying specific, practical routes to equity. Our findings and recommendations move beyond broad constructs into actions (what clinicians should say and do) to enable proactive inclusiveness.

Our study also extends prior findings to incorporate the perspectives of a broad age range of adult patients and their significant others living with varied serious illnesses and clinicians across multiple specialities. This increases the transferability and acceptability of our findings and recommendations beyond prior literature, which focused on specific illnesses^54^ or older people.^55^

### Implications

Inclusive, person-centred healthcare requires appropriate communication between service users and clinicians and organisational structures to support those interactions.^46^ Despite clinicians’ willingness to learn about LGBT+ inclusive care^56^ and evidence that doing so can improve knowledge and confidence,^57–59^ clinicians’ need for LGBT+ training persists.^60 61^

We provide evidence-based recommendations for LGBT+ inclusive care in the context of serious illness through simple communication strategies. Assessment and discussion in line with our recommendations will relieve pressure on patients and their significant others to determine whether sharing their LGBT+ identities is relevant. It will also reassure them that doing so will be met with understanding, care and respect. The full LGBT+ inclusive communication guide can be accessed here: www.kcl.ac.uk/nmpc/assets/research/projects/abc-lgbt-inclusive-communication.pdf

Our study recommendations may help to achieve national policy and monitoring standards (eg, NHS Sexual Orientation Monitoring Standard).^62^ While person-centred care was central to our participants and the recommendations we have made, delivering such care provides opportunities for improved monitoring data to inform broader understandings of health needs, outcomes and inequalities.^11 63^

### Future research directions

Our findings contribute to the evidence on person-centred care, offering practical and structural ways to integrate discussion of sexual orientation, gender identity and gender history into inclusive care. Evaluation of strategies to implement our recommendations are essential to understand effectiveness and broader challenges. Further research should focus on inclusive communication preferences of people who are bisexual and pansexual, transgender and non-binary, black, Asian and ethnic minorities, and those who live outside of urban centres.

Our evidence-based recommendations provide clear approaches for clinicians and organisations to reduce health inequality through proactive LGBT+ inclusive practice and systems.

## Data Availability

No data are available. To preserve the pseudonymity of the interviewees, the transcribed interviews are not available for sharing.
