# Supplementary material for: Communication about sexual orientation and gender between clinicians, LGBT+ people facing serious illness and their significant others: a qualitative interview study of experiences, preferences and recommendations
Source: BMJ Qual Saf. 2022 Aug 31;32(2):109–20. doi: 10.1136/bmjqs-2022-014792 (PMC9887369; doi:10.1136/bmjqs-2022-014792)
Supplement: Supplementary data [file bmjqs-2022-014792supp001.pdf]

Supplemental 1: ACCESSCare C Topic Guide – LGBT+ People (Patients)

Introductory Statement

Thank you for agreeing to participate in a research interview. I am one of a group of researchers from King's College London, and we are working on a research project which is looking to improve the way that sexual orientation, gender identity and gender history are discussed, addressed and documented in health care settings. As I will be asking you about sexual orientation, gender identity and gender history, as well as relationships with significant others, there may be some questions that you find sensitive. If there are any questions you would rather not answer then we can move on, or if you want to stop the interview at any time, just let me know. You may take a break and continue again or stop altogether. Everything you say in this interview will be confidential. If we use quotes for our research, we will make sure that you cannot be identified from the quote, by replacing names, places and any other identifiable information. Do you have any questions before we start?

Demographic information

- Age
- Gender / Trans Status / Gender History
  - "How do you describe your gender?"
  - "Sometimes, the sex you're assigned at birth, meaning on your original birth certificate, can be relevant in healthcare. Would you mind telling me what sex you were assigned at birth?"
  - "What are your pronouns?"
- Sexual Orientation
- Ethnicity (cultural ID)
- Relationship status (and duration)
- Living arrangement
- (If relevant: Gender of partner(s) /Trans status/ Gender history)

Illness History

- Diagnosis/diagnoses
- Time since diagnosis: when did you first become unwell?
- Can you tell me where you've received care for your illness?
  - (Prompt: Primary: GP/pharmacy/dentist/optician; Secondary: Hospital/Community, planned or emergency; Tertiary: highly specialised, referred from Secondary)
- What are your main needs and problems?
  - (Prompt: physical, psychological, social spiritual)
- How have those changed over time?

- Are you living with any other serious illnesses or conditions? (PROMPT: explore as above

- In what ways do you think your experience of your illness might be different because of your gender history?
- How about your experience of care, how might that have been different?
  - How did it make you feel?
  - Do you think that experience impacted on your feelings about healthcare afterwards?

- How about your sexual orientation, how has that impacted on your experience of illness and care?
  - How did it make you feel?
  - Do you think that experience impacted on your feelings about healthcare afterwards?

Preferred Approaches to Discussing Gender identity/gender history and Relationships

- When you are with your health care providers, so the doctors, nurses, and other members of the health care team, do any of them know your gender identity/gender history? What about the sex you were assigned at birth?
  - YES….how? OR NO….why?
- Is this how you prefer it to be?
- How do you feel about being asked directly about your gender identity/gender history?
- Should you be asked directly by the health care providers, or should it come from you first?
- Would you ever volunteer information about your gender identity/gender history without being asked?
  - When? Why? With whom?
  - How do you decide who to tell/not? Why?
  - What do you tell them?
- Have there been times when you have felt it was important to share your gender identity/gender history?
  - How do you decide when it's relevant?
- What kinds of things make it easier/less easy to let health care providers know your gender identity/gender history? (Prompt: communicative, environmental, societal, institutional)
- When taking your personal history or talking to you during appointments, do health care providers ever refer to your gender identity/gender history?
  - How do they do it? Well? Badly?
- Do you feel they respect and understand your gender identity/gender history? How do they demonstrate this?
- Do healthcare providers always use your correct pronouns?
- How do they react when they get your pronouns wrong?

- Are there phrases or words that the health care providers have used that made you feel more able to talk about your gender identity/gender history?
- How about in terms of the way they talk to you – their manner, body language?
- Do you find the health care providers use the same words to describe gender identity/gender history as you?
- Can you tell me about when it hasn't been handled well?

- Are there phrases or words used by the health care providers that made you feel uncomfortable about sharing your gender identity/gender history?
- Have there been times when their manner or body language have stopped you sharing your gender identity/gender history? How?
- How would you like your gender identity/gender history to be acknowledged and referred to by the health care providers?

Preferences and Concerns about Recording and Sharing Gender identity/gender history

- How do you feel about your gender identity/gender history being recorded in your health care records? How about the sex you were assigned at birth specifically? Or your pronouns?
- What do you see as the benefits of this information being documented?
  - Why?
- Would you have any concerns about this information being documented in health care records?
  - Why?
- How would you want that information to be shared with other healthcare professionals?
  - Why?

Preferred Approaches to Discussing Sexual Orientation and Relationships

- When you are with your health care providers, so the doctors, nurses, and other members of the health care team, do any of them know your sexual orientation? What about the gender or sex of your partner(s)?
    - YES….how? OR NO….why?
- Is this how you prefer it to be?
- How do you feel about being asked directly about these?
- Should you be asked directly by the health care providers, or should it come from you first?
- Would you ever volunteer information about your sexual orientation without being asked?
    - When? Why? With whom?
    - How do you decide who to tell/not? Why?
    - What do you tell them?
- Have there been times when you've felt it was important to share your sexual orientation?
    - How do you decide when it's relevant?
- What kinds of things make it easier/less easy to let health care providers know your sexual orientation? (PROMPT: communicative, environmental, societal, *institutional*)
- When taking your personal history or talking to you during appointments, do health care providers ever refer to your sexual orientation?
    - How do they do it? Well? Badly?
- Do you feel they respect and understand your sexual orientation? How do they demonstrate this?

- Are there phrases or words that the health care providers have used that made you feel more able to talk about your sexual orientation?
- How about in terms of the way they talk to you – their manner, body language?
- Do you find the health care providers use the same words to describe sexual orientation as you?
- Can you tell me about when it hasn't been handled well?

- Are there phrases or words used by the health care providers that made you feel uncomfortable about sharing your sexual orientation?
- Have there been times when their manner or body language have stopped you sharing your sexual orientation? How?
- How would you like your sexual orientation to be acknowledged and referred to by the health care providers?

Preferences and Concerns about Recording and Sharing Sexual Orientation and Relationships

- How do you feel about your sexual orientation being recorded in your health care records? How about the gender or sex of your partner(s)?
- What do you see as the benefits of this information being documented?
    - Why?
- Would you have any concerns about this information being documented in health care records?
    - Why?
- How would you want that information to be shared with other healthcare professionals?
    - Why?

Involvement of Partner/family/chosen family

- Do you ever take anyone with you to appointments?
    - Who do you take? How do you introduce them? Do healthcare professionals ask?
- *Do you want them present?*
- Have you felt they would be welcome?

  - Why? Why not?
- Can you tell me how they are acknowledged by health care providers?
- Have they talked about what the experience was like for them?
- Would you like it to be any different? How?
- How involved is your partner in decisions about your care?
- How involved is your family in these decisions?
- Who is listed as your Next of Kin? Is your partner listed? (if applicable)
  - When did you make that decision? Why was that important to you? Why not?

Training Recommendations

From our previous work, many health care professionals would like more guidance on discussing gender identity/gender history and relationship. From your experience, how could healthcare professionals improve the way they discuss or raise the subject of gender identity/gender history?

- How could healthcare professionals improve the way they discuss or raise the subject of sexual orientation?
- What recommendations would you make to healthcare professionals caring for LGBT people facing serious illness?
- How would you recommend they ask about gender identity/gender history?
  - What words would you suggest?
- How would you recommend they ask about pronouns?
  - What words would you suggest?
  - How should they react when they get pronouns wrong?

- How would you recommend they ask about sexual orientation?
  - What words would you suggest?
- How would you recommend they ask about significant others and important relationships?
  - What words would you suggest?

- When would you recommend these discussions be carried out?

- Are there any particularly good experiences you can recall that we could share as examples of good practice?
- How about more negative experiences, is there anything we should encourage professionals to avoid?

- Is there anything else you would recommend?
- Is there anything else you would like to add?
- Is there anything you would like to ask me about the study?

Thank participant for their time
