# Supplementary material for: Communication about sexual orientation and gender between clinicians, LGBT+ people facing serious illness and their significant others: a qualitative interview study of experiences, preferences and recommendations
Source: BMJ Qual Saf. 2022 Aug 31;32(2):109–20. doi: 10.1136/bmjqs-2022-014792 (PMC9887369; doi:10.1136/bmjqs-2022-014792)
Supplement: Supplementary data [file bmjqs-2022-014792supp002.pdf]

Supplemental 2: Additional quotes from stakeholders

Subtheme	Supporting quote
**Theme 1: Creating positive first impressions and building rapport**	
Creating first impressions and building rapport	"I always have that feeling where I can't decide how much I want to say […] when I was having this radiation test, the nurse kind of went through these questions and sort of asked when I had my last period and I just said that I don't have periods and she kind of looked a bit confused, and was like, 'Is that because of your contraception?' And this is the point where it depends, if it is a thing that's in passing that I can't be bothered to deal with, like I will often say to someone 'Oh it's because I don't have a womb.'" (Patient, queer non-binary woman, transgender, in their 30s)
Using neutral language	"we have to have people—, *get* people experienced in approaching the word 'partner.' So, if somebody says, if you came to me as a patient and you said, 'My partner,' I would always say, 'And what do *they* do?' *until* it's declared." (Doctor, in his 40s)
Listening and echoing terminology	"always referring to [my partner] as '*she*' all the time, even though I'm just like '*they*.' And they don't get it. Because they don't ask. But they keep doing it, and then it's really hard for me" (Patient, gay genderqueer person, assigned female at birth, in their 20s, with a non-binary partner)
Considering non-verbal signals	"It's like a physical repulsion, they physically back away from you […] And maybe it's subconscious, I'm sure people don't intend to do it. But you know, you know it's the thing that I'm gay but I don't fancy every woman. […] Like I've had people washing me when I was in intensive care and they're like—. I mean I can't physically move, but you won't wash me properly because you're scared I'm gonna get turned on, and I've had that said to me and you just think– it's just ridiculous, but *again* you just have to deal with it […] Because *what* can I do, in that situation." (Patient, gay female, cisgender, in her 20s)
**Theme 2: Enhancing care by actively exploring and explaining the relevance of sexual orientation and gender identity**	
Views on actively exploring and explaining the relevance of sexual orientation and gender identity	"Well as far as I'm concerned, issues of sexuality are completely *irrelevant* to the treatment you're receiving, so it's neither here not there. If it comes up and they know that you're…that's your social environment as it were, then that's fine, but really it should make no difference at all." (Patient, gay male, cisgender, in his 50s)
	"when I was younger, I had to hide it quite a lot and it would really embarrass people […] not even that long ago, even when I go to GPs the amount of embarrassment that I've somehow managed to cause just by saying that I'm gay is, is quite surprising. So I would tend, unless I feel that I need to, I'll probably just not answer or not go into it […] it might be me, my interpretation of things because of how it used to be rather than how it is now. I hope that's true, but yeah. See I'm getting embarrassed talking about it now which is crazy." (Significant other, bisexual female, cisgender, in her 50s)
	"what I would prefer is that people knew, […] it's just another, another facet of this person that makes up the whole person. It's not the be all and end all but it's a bloody big part" (Patient, gay male, cisgender, in his 50s)
	"I would find that, a bit you know 'Ooh what does that have to do with me, you know with my illness?' I don't think that would be the first question– If that was the first question that come out of their mouth and I would say, I'd be taken aback you know 'Why you need to know that?'" (Patient, gay male, cisgender, in his 60s)
	"I think I kind of only feel comfortable bringing it up with someone if I do feel comfortable around them, and I have a consultant at the hospital but to be honest I rarely ever see him because he's so busy and whenever I've got an appointment with him, normally it ends up being with somebody else and he doesn't actually come to the appointment" (Patient, queer non-binary person, assigned female at birth, in their 20s)
	"we're doing lots of work on sort of equal opportunities where you just treat everyone the same. Well you don't, you treat everyone according to their needs don't you. And you can't acknowledge that people might have different needs if you don't acknowledge difference can you. […] being blind to all these things doesn't actually make it better does it, sweeping things under the carpet just means they are not addressed." (Social worker, in her 40s)
	"you're kind of locked into this longstanding lie because you've either not brought it up or you've just gone along with someone's supposition that you have a different partner. It's harder then to break because you not only have to come out, also disprove their concepts about you. But also, defend that

	and it's—, yeah, it's just so much harder to be like, 'Actually, for the last year you thought this about me, but now I need to tell you that all that's untrue and this is actually the way I live my life.' And that can also become a barrier because, it goes on for so long." (Patient, gender-fluid gay person, assigned male at birth, in their 30s)
Communicating relevance of LGBT+ matters as part of quality, person-centred care	"it's not just me that reads their notes and it may be that although I've got the relationship with them that I may well know […] that other people don't know and, it's not fair to [pause] *out* them to everyone, if they don't want them to know. […] if I *had* to write in notes for any reason about it, I would always ask them." (Nurse, in his 20s)
	"I think the health professional has to point out that 'It's a safe space and everything that is discussed, is confidential and whilst I'd like to put it in the notes because it would be helpful, if you don't want it putting in the notes, I'll keep it up here [in my head].'" (Patient, gay male, cisgender, in his 70s)
	"it should be somebody's choice that it's recorded. […] I think it's a good idea, for me, but everybody else probably wouldn't feel the same way, or they'd feel labelled, targeted, or get paranoid about it." (Significant other, gay male, cisgender, in his 50s)
	"So, if somebody says, 'Are you married?' 'No. What is it to you? You know, why do you need to know? What does this have to do with my continuing healthcare? Why do you ask?' So, I should be able to say, 'Why do you ask?' without it being a big deal. 'Oh, we need to know because—' you know. It's all I want. I think that's fair really. So, now, 'Sure go ahead.' But why? […] I need the safety. 'What are you going to do with that information? How's it going to affect me? And, you know, my future.'" (Patient, gender non-conforming lesbian, assigned female at birth, in her 60s)
Including significant others and sexual orientation appropriately	"I mean *I* started having counselling myself around that time because I'd be…I was supporting her. […] I talk to her every morning on the way to work on the bus, I call her from the bus, that's when we have our chat, so that was like supporting her *emotionally*." (Significant other, female with fluid sexual orientation, cisgender, in her 40s)
	"if they were a bisexual man and they were with a man I would put them as a gay man, if they were a man and they came with a woman I would call them a heterosexual man. Terrible isn't it? I never even think about bisexual, and that is probably an issue. Well it is isn't it? Bisexual invisibility, people get very [pause] frustrated about […] I don't think a bisexual person would ever come up in conversation, it would just be, but then neither would, you see, neither does being a lesbian or a gay person when you're single, you know that equally becomes undefined" (Doctor, in her 40s)
Considering the environment and who else is present	"when they're going to open the curtains and walk off and you're thinking am I going to be judged by these people? Are they suddenly going to start kicking up a fuss saying I shouldn't be on a male ward? […] outing people can be quite dangerous, you don't know who else is on the ward. You could have someone who's really, really is against trans people, or their partner is and you know, you could just [pause] yeah get in quite a bit of trouble with people" (Patient, pansexual male, transgender, in his 20s)
	"I'd always want to ask permission to talk in front of that person as well. And if they are your neighbour or someone you know from work it may not be a good thing to do. So I always sort of routinely ask, you know 'I want to have an in-depth conversation. Is it alright if I do that with both of you or would you prefer me to come back another time?' I think those things are quite important" (Social worker, in her 40s)
	"a lot of trans people don't like– You know yes they are transgender from a medical point of view but as far as they're concerned that's not a part of their identity, like they're not trans. And I mean for me I'm, apart from in a medical setting, with you know people who knew me before hand, people like my colleagues and my friends don't know that I'm trans" (Patient, pansexual male, transgender, in his 20s)
**Theme 3: Visible and consistent LGBT+ inclusiveness in care systems**	
Standardising the approach to LGBT+ related discussions	"I mean sometimes we've supported people in gay relationships but they've maybe not said if it's been a little while ago, and we think they were in a same sex relationship but they spoke about the person as friend so we, we had to kind of go with that, that was the friend. I think there might be maybe something around generational, yeah, I'm not sure […] I mean, err the lady was older, and I think, I might be absolutely wrong here but I wondered if it was, you know she'd never come out as gay." (Social worker, in her 50s)
	"I think that I'd probably be apprehensive if I knew there was quite a like a really big *religious* vibe on the ward or something. D'you know what I mean. Or even just really religious staff because you don't really know-, 'cause obviously for a lot of people, you know, religion is like a *huge, huge,* you know

	driving force and factor in giving *meaning* to their life and stuff. And like, you know [pause] that *does* cause friction, you know, it *can* cause friction. I haven't *actually* experienced it but, you know, you just—, you'd have to make that call. You'd have to make a generalisation against someone else, wouldn't you? Which is the *same thing*." (Patient, gay genderqueer person, assigned female at birth, in their 20s)
	"I think I could ask a young white British person that question a lot more easily than a 65-year-old, black Nigerian man. Or I might feel like I know what the answer's gonna be more for him, but I don't actually have any reason to think that […] it just feels like I could really offend him for suggesting that it's not clear he's straight, and so I probably avoid it more in groups where it feels like the question will be less tolerated." (Doctor, in his 30s)
	"I think the way it's gone about, it needs to be standardised or just be in a *particular* way, that no one gets worried and no one gets offended. It's just very general and it's very particular. The more you *flounder* around a scenario or work out how nice it is to say, it just makes the whole thing seem awkward. You just need to be (laughter) quite standard with it and just ask direct. And people don't mind. […] Just make it more *formal* about how, how you're asking the information. (Significant other, gay male, cisgender, in his 40s)
	"you don't want to target specific cohort of patients because of an assumption that you might have, and that's not a right thing. 'Cause I think we need to ask *all* patients […] I think most practitioners tend to follow templates, if I can call it that, you know, assessment, or sort of engagement kind of tool. So, if that assessment or tool has that information, I think that you can ask and follow it through. […] even if the patient were to say, 'Why are you asking? Would you ask it to everybody else?' It's that element I think that a lot of practitioners would think, you know, why would you only ask a particular patient or a particular type of patient, as supposed to not everybody." (Social worker, in his 30s)
Establishing inclusive processes	"I think there are a lot of problems that come about because my records say that I'm female which means that I'm not, later on in life, I'm not gonna come up for prostate cancer screenings, just like trans men like suddenly don't come up for cervical cancer screening. And, actually like that is because medical records are full of assumptions which is just like I changed the gender marker from M to F and since that happened, it then gets immediately assumed that I have a womb, ovaries, cervix and don't have a prostate and testicles and that is a problem." (Patient, queer non-binary woman, transgender, in their 30s)
	"I feel like there's a lot of fear in regards to keeping records, because if you look historically, there was a reason to be fearful. And I think that's where it's the role of the healthcare practitioners to provide the confidence that everything is going to be treated with respect. You know, 'cause it's like—, it is something incredibly personal about you. You don't necessarily want everyone to know it. That kind of stuff. But that's why I think for chronic conditions and serious conditions, it's different. It's not like you stub your toe and like, 'Oh, do you have a boyfriend or girlfriend?', you know, it's just like, 'This is going to impact your life. Let's get an idea of what your life is.'" (Patient, gender-fluid gay person, assigned male at birth, in their 30s)
Markers of inclusiveness	"I know that (the hospital) have got the brilliant rainbow badges they— which are really really cool and I must confess I would very much like one but none the less it— So I think you know that type of thing is incredibly important because it just means you, you know for patients who may or may not have felt comfortable before, to turn around say 'Well hold on a minute here. You know these people are wearing a rainbow badge' or you know 'This clinician's wearing a rainbow badge. She's clearly, not necessarily that she gets what I'm on about, but she's clearly *aware* that this is something that can be talked about here' so actually it just makes me more approachable" (Nurse, in her 40s)
